# Investigating Gamma Frequency Band PSD in Alzheimer’s Disease Using qEEG from Eyes-Open and Eyes-Closed Resting States

**DOI:** 10.3390/jcm14124256

**Published:** 2025-06-15

**Authors:** Chanda Simfukwe, Seong Soo A. An, Young Chul Youn

**Affiliations:** 1 Department of Bionano Technology, Gachon University, Seongnam-si 1342, Republic of Korea; chandaelizabeth94@gmail.com; 2 Department of Neurology, College of Medicine, Chung-Ang University, Seoul 06974, Republic of Korea; 3 Department of Medical Informatics, College of Medicine, Chung-Ang University, Seoul 06974, Republic of Korea

**Keywords:** Alzheimer’s disease, aging, power spectra density, coherence, electroencephalography, gamma

## Abstract

**Background/Objectives:** Gamma oscillations (30–100 Hz), which are essential for memory, attention, and cortical synchronization, remain underexplored in Alzheimer’s disease (AD) research. While resting-state EEG studies have predominantly examined lower frequency bands (delta to beta), gamma activity may more accurately reflect early synaptic dysfunction and other mechanisms relevant to AD pathophysiology. AD is a common age-related neurodegenerative disorder frequently associated with altered resting-state EEG (rEEG) patterns. This study analyzed gamma power spectral density (PSD) during eyes-open (EOR) and eyes-closed (ECR) resting-state EEG in AD patients compared to cognitively normal (CN) individuals. **Methods**: rEEG data from 534 participants (269 CN, 265 AD) aged 40–90 were analyzed. Quantitative EEG (qEEG) analysis focused on the gamma band (30–100 Hz) using PSD estimation with the Welch method, coherence matrices, and coherence-based functional connectivity. Data preprocessing and analysis were performed using EEGLAB and Brainstorm in MATLAB R2024b. Group comparisons were conducted using ANOVA for unadjusted models and linear regression with age adjustment using log_10_-transformed PSD values in Python (version 3.13.2, 2025). **Results**: AD patients exhibited significantly elevated gamma PSD in frontal and temporal regions during EOR and ECR states compared to CN. During ECR, gamma PSD was markedly higher in the AD group (Mean = 0.0860 ± 0.0590) than CN (Mean = 0.0042 ± 0.0010), with a large effect size (Cohen’s *d* = 1.960, *p* < 0.001). Conversely, after adjusting for age, the group difference was no longer statistically significant (β = −0.0047, SE = 0.0054, *p* = 0.391), while age remained a significant predictor of gamma power (β = −0.0008, *p* = 0.019). Pairwise coherence matrix and coherence-based functional connectivity were increased in AD during ECR but decreased in EOR relative to CN. **Conclusions**: Gamma oscillatory activity in the 30–100 Hz range differed significantly between AD and CN individuals during resting-state EEG, particularly under ECR conditions. However, age-adjusted analyses revealed that these differences are not AD-specific, suggesting that gamma band changes may reflect aging-related processes more than disease effects. These findings contribute to the evolving understanding of gamma dynamics in dementia and support further investigation of gamma PSD as a potential, age-sensitive biomarker.

## 1. Introduction

Gamma oscillations (30–100 Hz) have gained significant attention in Alzheimer’s disease (AD) research due to their essential role in memory, attention, and perception, cognitive domains that are characteristically impaired in AD [1,2]. Therefore, the investigation of gamma activity has become a central focus in understanding the neurophysiological underpinnings of AD. These oscillations are closely linked to parvalbumin-expressing inhibitory interneurons [3], with animal studies demonstrating that enhancing interneuron function can restore gamma activity, reduce hypersynchrony, and improve cognition [4,5]. On the other hand, findings in AD remain inconsistent; while some studies report reduced gamma synchronization and power [4,5,6,7], others reported elevated gamma activity during resting-state EEG and cognitive tasks [8,9,10]. These conflicting results highlight unresolved questions surrounding gamma dynamics in AD pathophysiology.

Gamma alterations are also associated with aging. Healthy older adults typically exhibit lower peak gamma frequencies than younger individuals [11,12]. Notably, Güntekin et al. reported that AD patients show further reductions in dominant gamma peak frequency during both eyes-open (EOR) and eyes-closed (ECR) resting conditions compared to age-matched controls [13], suggesting overlapping effects of aging and disease. While the underlying mechanisms remain unclear, abnormal gamma activity is increasingly viewed as a promising biomarker and therapeutic target in cognitive disorders, such as AD [7,14,15].

These gamma-specific dynamics are best understood within the broader context of AD, the most prevalent form of dementia, which progressively impairs memory and cognition, primarily affecting individuals over 65 years [16]. The defining pathological features of AD include the buildup of amyloid β (Aβ) peptides, which form senile plaques and neurofibrillary tangles made up of hyperphosphorylated tau proteins [17]. These pathological alterations result in neuronal death and synaptic impairments, particularly within the hippocampus and cortical areas vital for cognitive processes [17,18], which lead to changes in neuronal activity commonly observed in patients with AD [19,20]. Electroencephalography (EEG), a non-invasive and cost-effective method with high temporal resolution, is used as a biomarker for the early detection and diagnosis of AD through quantitative EEG (qEEG) analysis [21,22,23]. Studies often utilize resting-state EEG (rEEG) recorded under both eyes-open resting (EOR) and eyes-closed resting (ECR) conditions, primarily to gain insights into neural network activity during both healthy and pathological aging processes, where subjects are awake but not engaged in specific tasks [13,24,25].

In rEEG studies, AD is associated with a deceleration of EEG rhythms, marked by a decrease in power within higher frequency bands (alpha 8–13 Hz and beta 13–30 Hz) and an increase in power within lower frequency bands (delta 0.5–4 and theta 4–8) [26,27]. Mild cognitive impairment (MCI), which is considered an intermediate stage between normal aging and dementia, also demonstrates EEG changes, though these are less pronounced compared to AD [28]. Nevertheless, gamma band dynamics during rest remain poorly characterized, despite their growing relevance.

To address this gap, we investigated gamma band power spectral density (PSD) during resting-state EEG in AD and cognitively normal (CN) elderly individuals. We hypothesized that the PSD within the gamma frequency band would exhibit distinct differences between AD patients and CN, with AD patients expected to demonstrate relatively lower gamma frequencies. To evaluate this hypothesis, we conducted a retrospective analysis of rEEG recordings from our database, including data from AD patients and CN. The primary objective of this study was to address the existing gap in the literature regarding the PSD of gamma frequency properties in resting-state EEG among CN and AD patients. We anticipate that these findings will serve as foundational data for future research on gamma frequency, particularly in identifying the optimal frequency for therapeutic interventions and biomarkers for AD patients.

## 2. Methods

### 2.1. Demographics of the Participants

The EEG signals from a total of 534 participants, comprising CN (*n* = 269) and AD (*n* = 265) aged 40–90 years, were analyzed. The average ages of the CN and AD groups were 64.54 ± 9.03 and 76.94 ± 8.03 years, respectively (mean ± standard deviation). Data collection was approved by the Chung-Ang University Hospital Institutional Review Board and the ethics committee (IRB Approval No. 2009-005-19331). This hospital-based cohort study was designed to assess the prevalence of cognitive impairments and related risk factors in elderly individuals, in accordance with the ethical standards of the Declaration of Helsinki. All participants provided written informed consent prior to their inclusion in the study.

The selection criteria for the CN group aligned with the 28 normal elderly criteria established by Christensen et al. [29]. The CN participants were required to have a Korean Mini-Mental State Examination (K-MMSE) score of −1 standard deviation (SD) or higher, a Korean-Instrumental Activities of Daily Living (K-IADL) score of 0.42 or less, and a Korean Dementia Screening Questionnaire (KDSQ) score of 6 or lower [30,31]. Additionally, all CN participants underwent MRI to confirm the absence of structural abnormalities, such as cerebrovascular lesions or significant atrophy, ensuring that they met the criteria for normal cognitive aging.

The diagnostic criteria for AD subjects included clinically evident progressive memory decline, diminished ability to perform activities of daily living, and personality changes, along with objective verbal memory deficits assessed using the Seoul Neuropsychological Screening Battery (SNSB) [32,33]. The SNSB, a comprehensive neuropsychological test developed in Korea, evaluates multiple cognitive domains, including memory, attention, language, executive function, and visuospatial processing (Supplementary Table S1). Additionally, MRI was utilized to rule out structural anomalies, ensuring that observed cognitive deficits were attributable to AD pathology rather than confounding neuropathology. All diagnoses were conducted by a neurologist following neuropsychological assessment and established diagnostic criteria [33].

### 2.2. EEG Signals and Preprocessing

The EEG data were recorded using a Comet AS40 amplifier system (GRASS; Telefactor, Conshohocken, PA, USA) in conjunction with gold-cup electrodes. Electrode positioning followed the international 10–20 system, resulting in a total of 19 electrodes placed at the following locations: Fp1, Fp2, F7, F3, Fz, F4, F8, T3, C3, Cz, C4, T4, T5, P3, Pz, P4, T6, O1, and O2, with earlobes designated as the reference sites (see Supplementary Figure S1). To maintain high signal fidelity, electrode–skin impedance was carefully monitored and consistently kept below 5 kΩ during the entire recording process, and participants were seated in a quiet, controlled environment and instructed to remain relaxed and still, minimizing movements, such as blinking or swallowing, to reduce potential artifacts during the recording sessions.

The EEG signals were recorded digitally and safely saved on magnetic disks. Prior to acquisition, digital signal processing techniques were applied, including bandpass filtering to focus on the desired frequency range while minimizing noise and unrelated signals. A frequency band of 0.5–100 Hz was chosen to retain relevant brain activity and eliminate potential artifacts. During the sessions, participants alternated between EOR and ECR states. The data were sampled at 200 Hz, with 10 trials per condition, each lasting 30 s, resulting in approximately 5 min of EEG data for both EOR and ECR conditions. After preprocessing and artifact rejection, a minimum of 60 s of clean data per condition was retained for each subject to ensure spectral reliability. On average, 45 artifact-free 4 s epochs (approximately 180 s) were included for each participant per condition.

**Figure 1:**
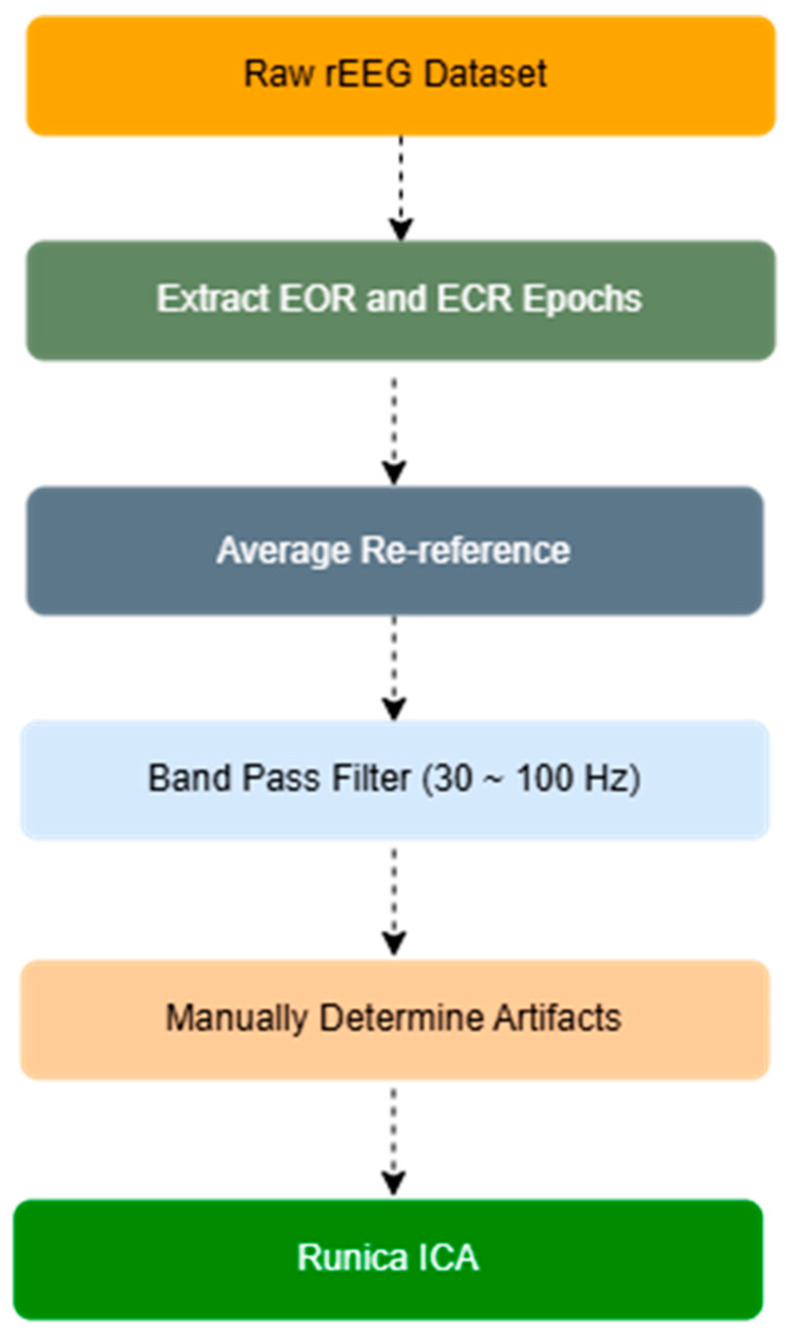
A schematic illustration outlining the preprocessing workflow and artifact removal procedures implemented for the rEEG signals. Abbreviations: rEEG, resting state electroencephalography; EOR, eyes-open resting state; ECR, eyes-closed resting state; ICA, independent component analysis.

The raw EEG data were processed using the EEGLAB toolbox (version 2024) in the MATLAB environment (R2024a; http://www.sccn.ucsd.edu/eeglab/ (14 February 2025)). Preprocessing followed standard procedures, including signal amplification, bandpass filtering, removal of artifacts, and application of independent component analysis (ICA). As part of this pipeline, average re-referencing was applied prior to ICA to reduce channel-wise baseline variance, leveraging the uniform electrode montage and consistently high signal quality across participants. This step, though not standard in all contexts, is supported within the EEGLAB framework and has been employed in previous studies under similar conditions [34]. These procedures were executed following a structured workflow, outlined in the block diagram presented in Figure 1, to ensure the extracted relative PSD data were free of artifacts and suitable for analysis.

### 2.3. Spectral and Functional Connectivity Analysis

PSD analysis was performed using the Brainstorm toolbox version 2022 [35] within the MATLAB environment. EEG recordings were first preprocessed using standard procedures, as illustrated in Figure 1. Continuous data were segmented into epochs corresponding to EOR and ECR conditions. PSD was estimated using Welch’s method [36], employing a Hamming window of 1 s with 50% overlap. PSD values were computed for each electrode and averaged across epochs. The resulting spectral estimates were expressed in decibels (dB), and gamma band power (30–100 Hz) was extracted for comparison between the EOR and ECR conditions. All processed PSD values were exported in “.csv” format for subsequent statistical analysis (Supplementary Table S2). Summary PSD results are presented in Table 1.

Coherence analysis was performed in Brainstorm using magnitude-squared coherence between electrode pairs within the 30–100 Hz gamma band, using 1-s windows with 50% overlap. Coherence values were averaged across epochs to generate pairwise connectivity matrices). To visualize spatial distribution, topographic scalp maps of gamma power were created by averaging PSD values across epochs and projecting them onto a 2D head model using spherical interpolation. A consistent color scale was applied across all subjects and conditions, with warmer colors representing higher gamma power, allowing for direct visual comparison. Functional connectivity was assessed from the coherence matrices, generating 19 × 19 adjacency matrices for each participant. A fixed threshold (coherence ≥ 0.8) was applied to highlight the strongest connections for group-level visualization and interpretation.

### 2.4. Statistical Analysis

**Figure 2:**
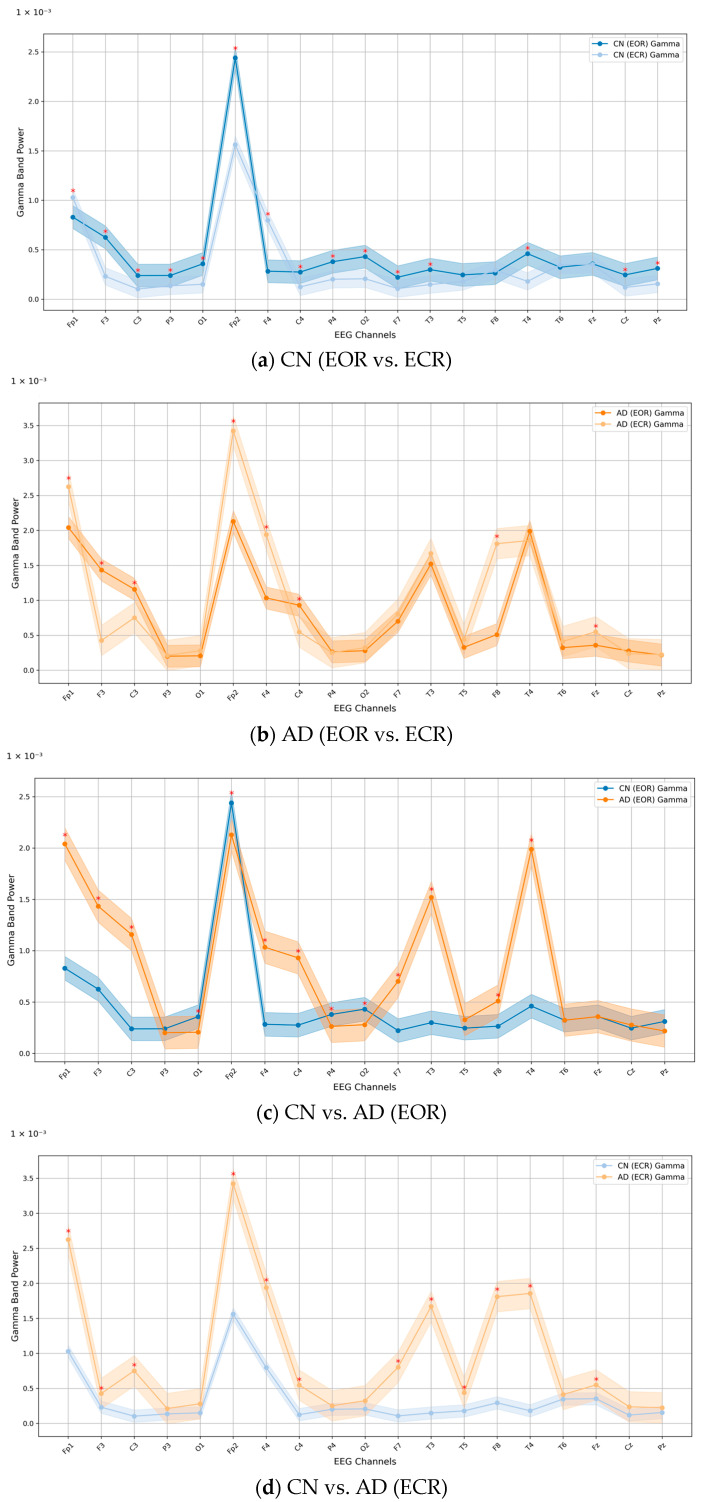
The overall PSD of 19 EEG channels in the gamma band for (**a**) CN (EOR vs. ECR), (**b**) AD (EOR vs. ECR), (**c**) CN vs. AD (EOR), and (**d**) CN vs. AD (ECR). Note: The shaded areas around each line represent the standard error of the mean (SEM), illustrating the variability and reliability of the group average. Red asterisks (*) indicate EEG channels where the effect size, calculated using Cohen’s *d*, was large (*d* ≥ 0.8), reflecting meaningful differences in gamma band power between the compared groups. Abbreviations: PSD, power spectrum density; EEG, electroencephalography; CN, healthy controls; AD, Alzheimer’s disease; EOR, eyes-open resting state; ECR, eyes-closed resting state.

**Figure 3:**
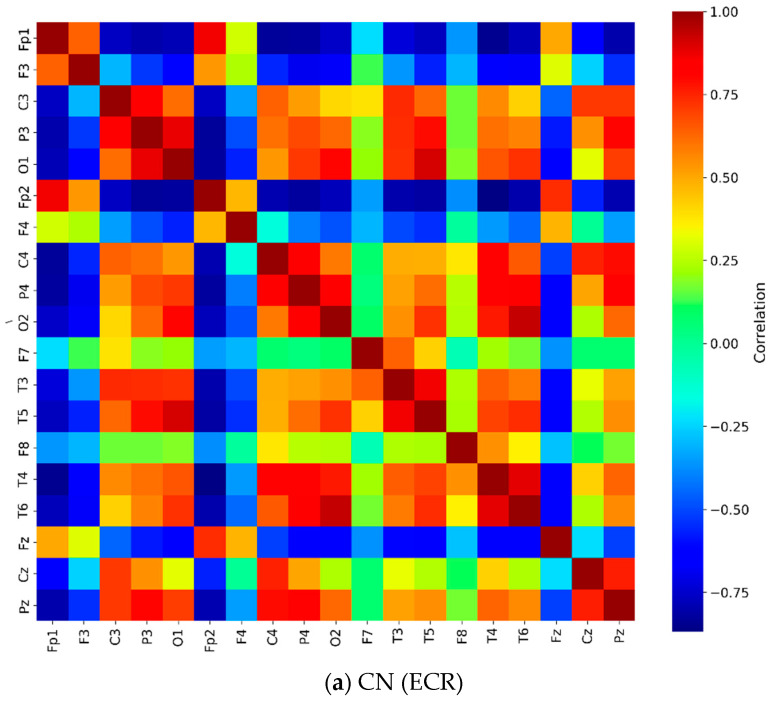

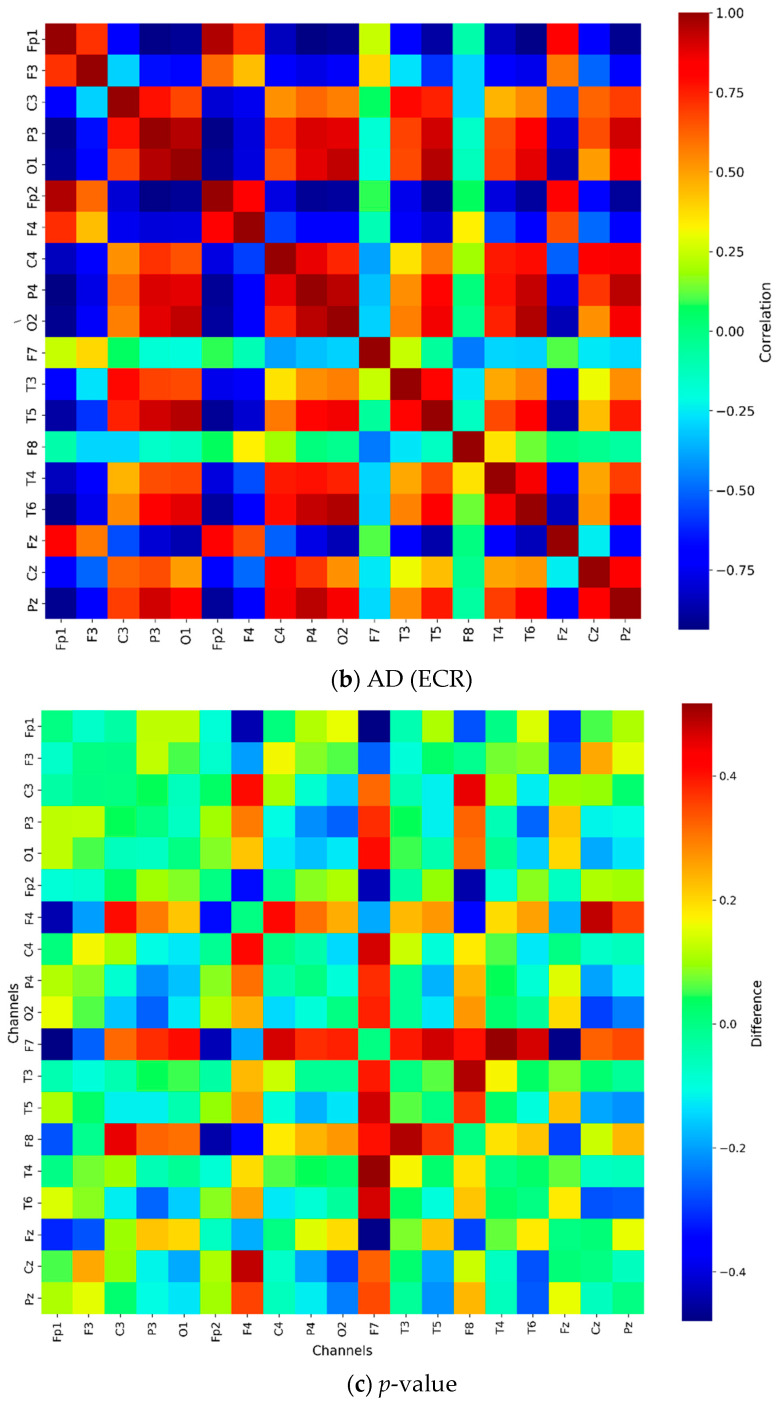
Coherence matrix for pairwise electrodes in the gamma band for (**a**) the CN group during the ECR condition and (**b**) the AD group during the ECR condition. (**c**) The *p*-value matrix (*p* < 0.001) obtained from ANOVA with Bonferroni correction, comparing the two groups. Diagonal entries are highlighted in red to indicate the most statistically significant values. Notes: The color scale shows coherence differences between CN and AD during the ECR condition. Red/yellow indicates higher coherence in CN; blue indicates higher coherence in AD. Only Bonferroni-corrected significant differences (*p* < 0.05) are displayed. Abbreviations: CN, healthy controls; AD, Alzheimer’s disease; ECR, eyes-closed resting state; *p*-value, probability; ANOVA, analysis of variance.

All analyses were conducted using Python (version 3.13.2, 2025) within the PyCharm Integrated Development Environment (IDE) (JetBrains, Amsterdam, The Netherlands, https://www.jetbrains.com/pycharm/ (15 March 2025)). Group-level comparisons of PSD were first evaluated using a one-way analysis of variance (ANOVA) via the “f_oneway” function from the SciPy library (https://scipy.org/ (15 March 2025)). To further examine the influence of age as a potential confounder, we performed linear regression analyses using log_10_-transformed gamma PSD values, with age included as a covariate. These models were implemented using the “ols” function from the statsmodels library (https://www.statsmodels.org/ (15 March 2025)). Unadjusted comparisons were performed using raw PSD values, while log_10_-transformed PSD values were used for regression models to ensure the normality of residuals and stabilize the variance. Regression outputs included beta coefficients (β), standard errors (SE), and *p*-values, enabling a clearer interpretation of whether observed group-level differences persisted after controlling for age. To correct multiple comparisons in intra-group analyses (Figures 3c and 5c), Bonferroni-adjusted *p*-values were used. In contrast, for Figure 2, traditional statistical tests were insufficient due to the extremely low magnitude of gamma band power values; thus, we computed effect sizes using Cohen’s *d* [37] to assess practical significance. All tests were two-tailed, with significance thresholds set at *p* < 0.001 for unadjusted comparisons and *p* < 0.05 for age-adjusted models.

## 3. Results

### Relative PSD Analysis

Table 1 summarizes the within-group comparison of relative gamma band PSD between EOR and ECR conditions in the CN and AD groups. A statistically significant increase in gamma power was observed during the ECR condition compared to EOR in both groups (*p* < 0.001). In the CN group, the increase was minimal, as reflected by a small effect size (Cohen’s *d* = 0.0894). In contrast, the AD group demonstrated a pronounced increase in gamma PSD from EOR to ECR, with a large effect size (Cohen’s *d* = 1.9000), indicating a substantially heightened neural response at ECR resting in individuals with AD.

Table 2 presents both unadjusted and age-adjusted comparisons of gamma band power between CN and AD groups under EOR and ECR conditions. In the unadjusted analysis, a statistically significant difference in mean gamma PSD was observed between CN (0.0040 ± 0.0030) and AD (0.0060 ± 0.0080) during the EOR condition (*p* < 0.001; Cohen’s *d* = 0.331). A larger group difference emerged during the ECR condition, with CN showing a mean PSD of 0.0042 ± 0.0010 and AD showing 0.0860 ± 0.0590 (*p* < 0.001; Cohen’s *d* = 1.960). In the age-adjusted linear regression using log_10_-transformed gamma PSD, the group effect (CN vs. AD) was not statistically significant in either condition (EOR: β = −0.0022, SE = 0.0033, *p* = 0.504; ECR: β = −0.0047, SE = 0.0054, *p* = 0.391). In contrast, age was significantly associated with reduced gamma power during the ECR condition (β = −0.0008, SE = 0.0003, *p* = 0.019), suggesting that age-related decline may substantially account for the observed differences in gamma band activity rather than reflecting AD-specific effects. Figure 2 illustrates these differences across 19 EEG channels, showing spatial patterns of gamma PSD in CN and AD across both conditions. The gamma band PSD values ranged from 0 to approximately 3.5, indicating substantial variability in power distribution across the channels.

When comparing within-group conditions (Figure 2a,b), gamma PSD was generally higher during the ECR condition than EOR. In the CN group, large effect sizes (*d* > 0.8) were observed at Fp1, F3, C3, P3, O1, Fp2, F4, C4, P4, O2, F7, T3, T4, Cz, and Pz. In the AD group, ECR elicited markedly higher gamma activity, with large effect sizes at Fp1, Fp3, F3, F4, T3, T4, and F8. Between-group comparisons (Figure 2c,d) revealed distinct patterns in gamma PSD. For the EOR condition, electrodes showing large effect sizes (*d* > 0.8) between CN and AD groups included Fp1, F3, C3, O1, Fp2, F4, C4, P4, O2, F7, T3, F8, and T4. Under the ECR condition, large effect sizes were observed at Fp1, F3, C3, Fp2, F4, C4, F7, T3, T5, F8, T4, and Fz. These results highlight substantial differences between groups, particularly across frontal and temporal regions.

Coherence measures normalized linear synchronization between EEG signals analyzed in the frequency domain. This study performed coherence analysis on combinations of EEG channel pairs for both CN and AD groups. The relative PSD in the ECR condition demonstrated highly significant differences between the two groups (CN vs. AD). The average coherence across 19 channels in the gamma frequency band during the ECR condition is presented in Figure 3. The symmetrical distribution of mean coherence values in both groups reflects consistent synchronization patterns across scalp-recorded channels. The coherence matrix displayed a diagonal with all entries equal to 1, indicating perfect synchronization of an EEG signal with itself.

**Figure 4:**
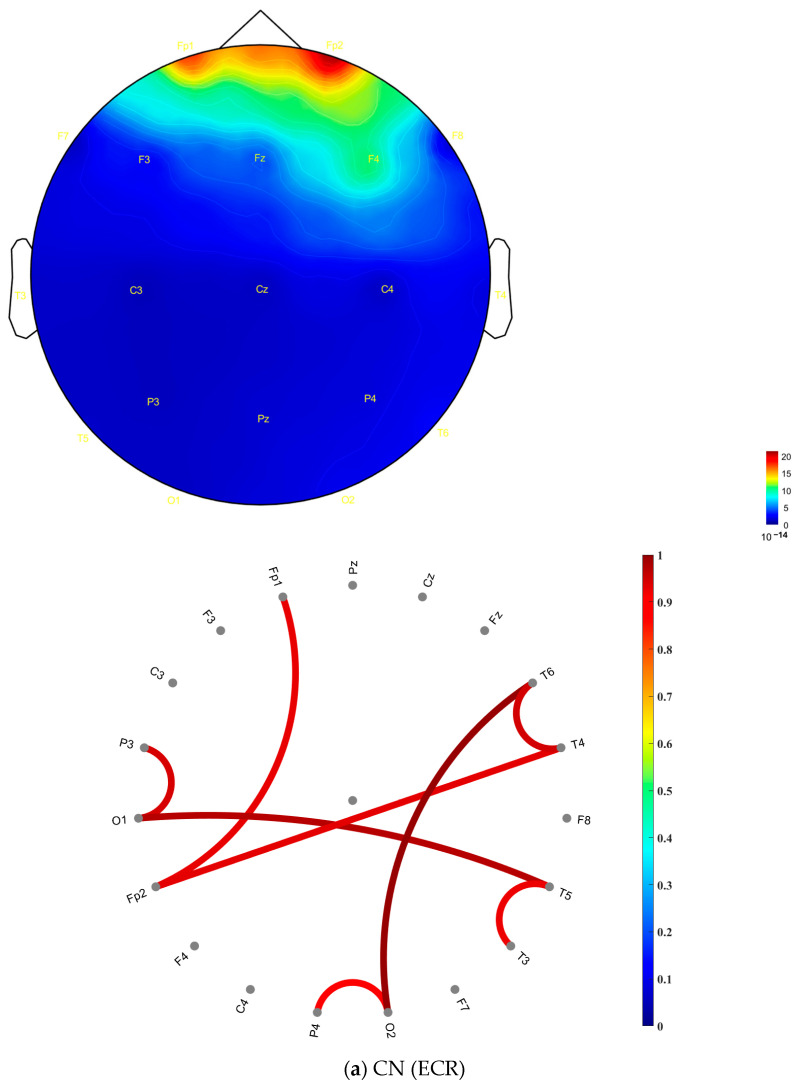

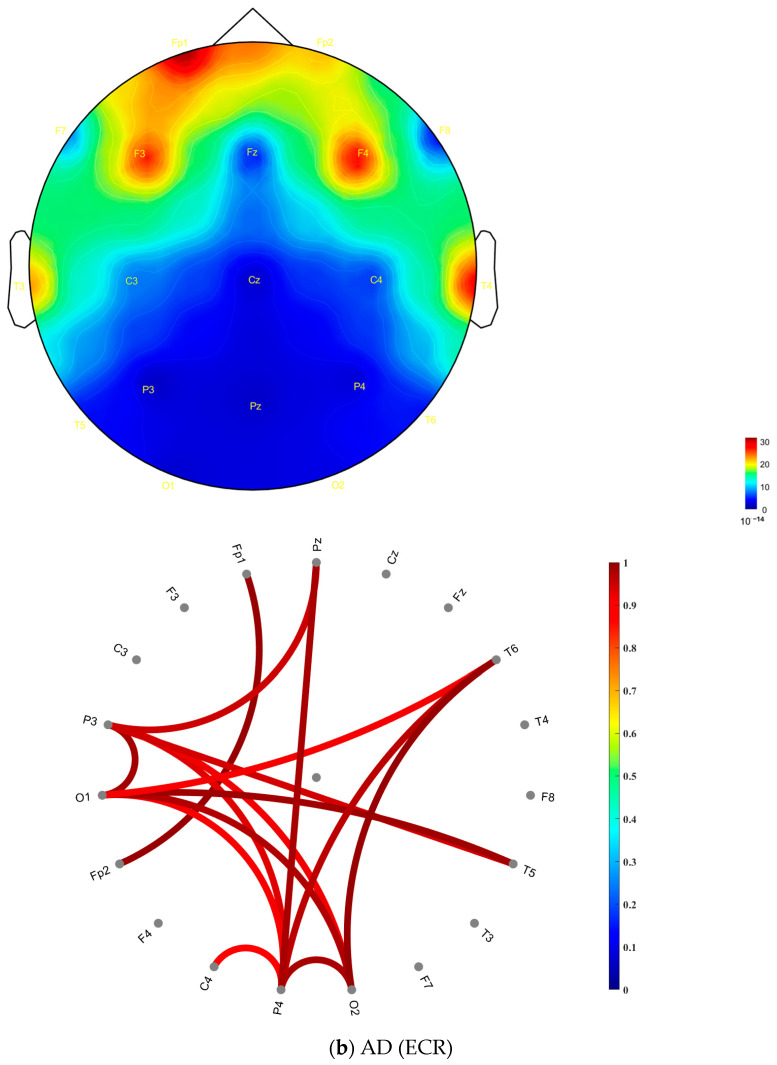
Topographic distributions and coherence-based functional connectivity in the gamma frequency band for (**a**) CN (ECR) and (**b**) AD (ECR) across 19 EEG channels. The left panels depict scalp topographies of gamma PSD in units of 10^−14^, while the right panels illustrate functional connectivity based on pairwise EEG channel coherence. Note: Color bars indicate the value scale for each plot type. PSD values are in the order of 10^−14^ (shown in the topographic maps), while coherence values range from 0 to 1 (shown in the connectivity plots). Only coherence values ≥ 0.8 are displayed to highlight strong neural synchrony. Red colors indicate higher gamma power or stronger coherence. The 0.8 threshold is used for visualization and does not imply statistical significance. Abbreviations: EEG, electroencephalography; CN, healthy controls; AD, Alzheimer’s disease; ECR, eyes-closed resting state.

In the AD group (Figure 3b), more regions exhibited high coherence values (indicated by red coloring) compared to the CN group. In contrast, the CN group (Figure 3a) showed high coherence values that were more diffusely distributed across frontal, central, occipital, parietal, and temporal areas within the gamma frequency band. The distribution of these high coherence values was more scattered and less concentrated than that of the AD group. Figure 3c presents the matrix of *p*-values obtained from the ANOVA analysis, assessing coherence differences between the CN and AD groups during the ECR condition. This matrix was derived by computing the statistical differences between the coherence values of the CN group (Figure 3a) and the AD group (Figure 3b). The consistently low *p*-values (*p* < 0.001) indicate highly significant differences in coherence patterns between the two groups. Figure 4 presents topographic maps and corresponding connectivity diagrams for both groups. The topographic maps illustrate the spatial distribution of PSD, while the red links in the connectivity diagrams represent high coherence values between electrodes, highlighting functional connections.

In the CN group (ECR), Figure 4a, the topographic map, shows a relatively uniform PSD distribution across the scalp, with higher PSD values localized around the frontal and central regions, particularly at F3, F4, and Fz, with widespread and balanced functional connections across electrode sites. This suggests a well-distributed synchronization and efficient information flow between brain regions, which are characteristics of healthy neural networks.

In contrast, the AD group (ECR) in Figure 4b exhibits a different pattern. The topographic map reveals elevated PSD values in the frontal, parietal, and temporal regions, particularly at Fp1, Fp2, T6, T4, and P4. The connectivity diagram highlights stronger and more localized strong electrode-level coherence values predominantly observed between specific electrodes within these regions. This increased local connectivity, coupled with fewer inter-regional connections, suggests a disruption in the global integration of brain networks. The imbalance in synchronization likely reflects impaired information transmission and reduced collaborative interactions across broader brain regions, which are hallmark features of neurodegeneration in AD.

## 4. Discussion

This study investigated changes in PSD within the gamma frequency band (30–100 Hz) in AD patients (*n* = 265) compared to CN (*n* = 269) using rEEG recordings. Previous research has highlighted that PSD alterations of the gamma band in rEEG can offer valuable insights into the functional disruptions associated with AD [9,38,39]. Frequency domain analysis has been extensively employed to examine these changes, complemented by coherence analysis, which quantifies the degree of synchronization between specific brain cortical regions [40,41].

**Figure 5:**
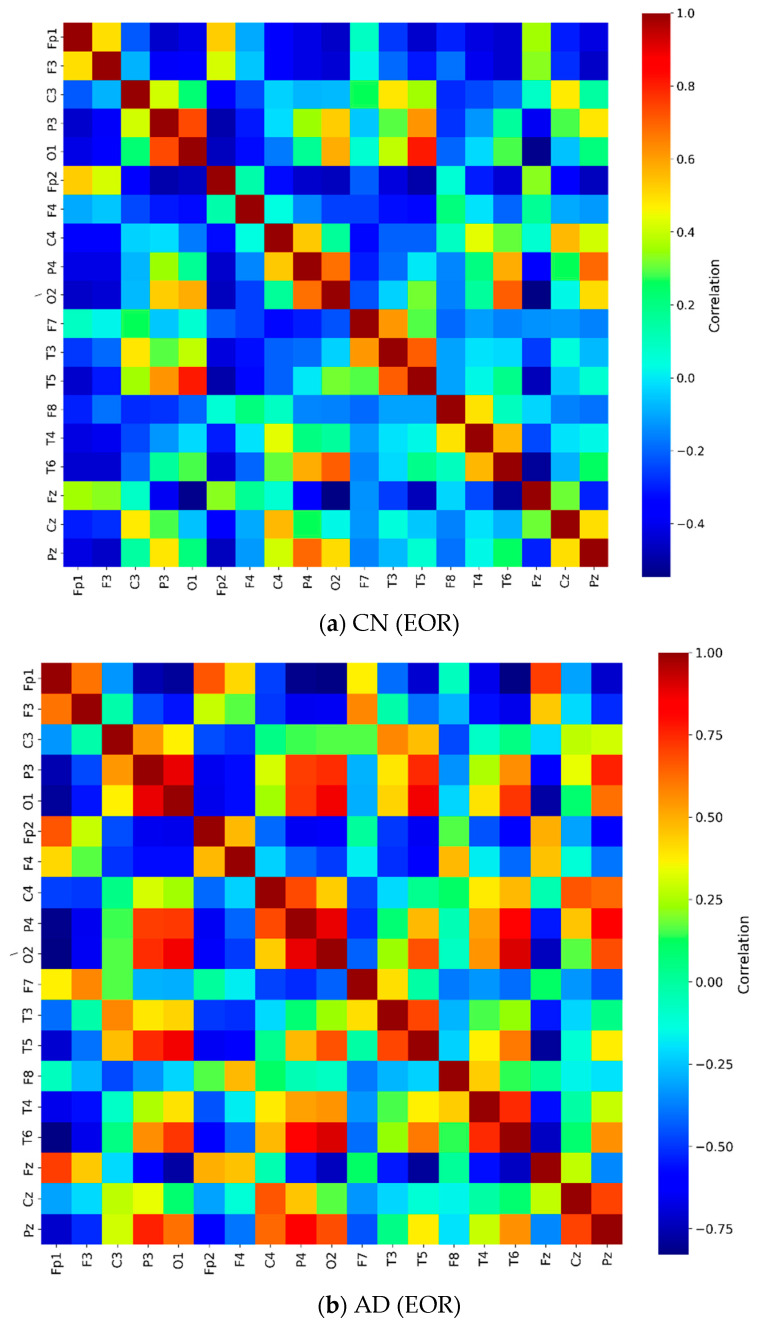

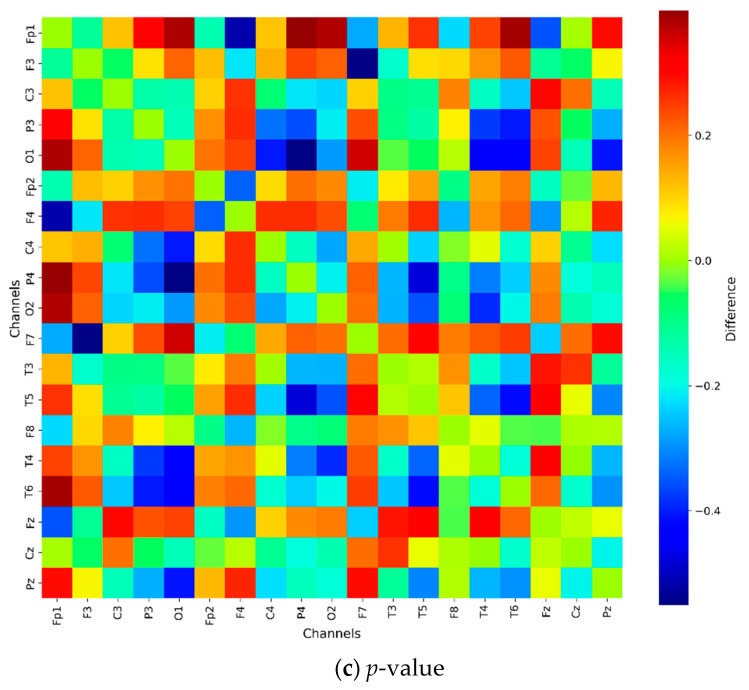
Coherence matrix for pairwise electrodes in the gamma band for (**a**) the CN group during the EOR condition and (**b**) the AD group during the EOR condition. (**c**) The *p*-value matrix (*p* < 0.001) obtained from ANOVA with Bonferroni correction, comparing the two groups. Diagonal entries are highlighted in red to indicate the most statistically significant values. Notes: The color scale shows coherence differences between CN and AD during the ECR condition. Red/yellow indicates higher coherence in CN; blue indicates higher coherence in AD. Only Bonferroni-corrected significant differences (*p* < 0.05) are displayed. Abbreviations: CN, healthy controls; AD, Alzheimer’s disease; EOR, eyes-open resting state; *p*-value, probability; ANOVA, analysis of variance.

**Figure 6:**
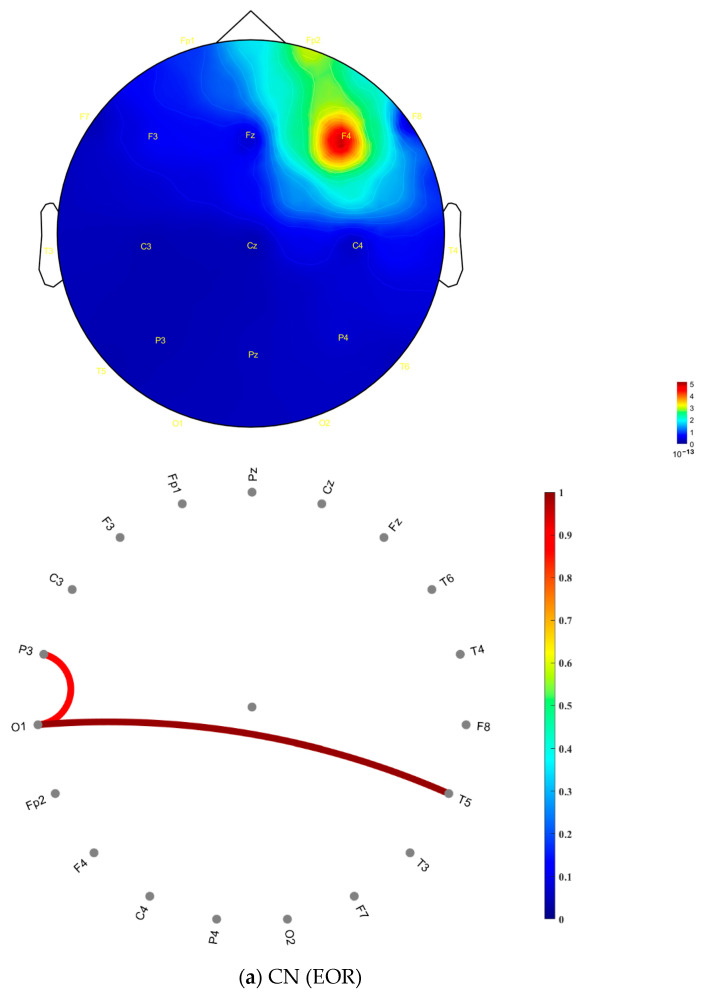

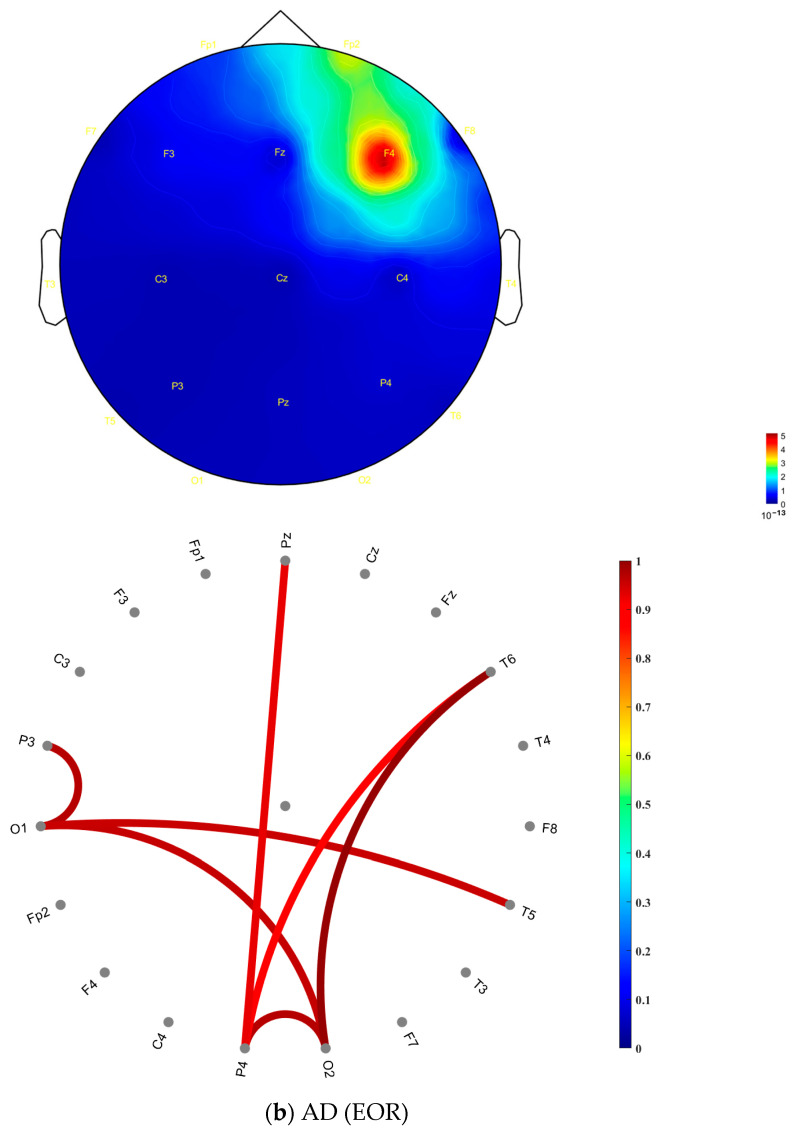
Topographic distributions and coherence-based functional connectivity in the gamma frequency band for (**a**) CN (EOR) and (**b**) AD (EOR) across 19 EEG channels. The left panels depict scalp topographies of gamma PSD in units of 10^−14^, while the right panels illustrate functional connectivity based on pairwise EEG channel coherence. Note: Color bars indicate the value scale for each plot type. PSD values are in the order of 10^−14^ (shown in the topographic maps), while coherence values range from 0 to 1 (shown in the connectivity plots). Only coherence values ≥ 0.8 are displayed to highlight strong neural synchrony. Red colors indicate higher gamma power or stronger coherence. The 0.8 threshold is used for visualization and does not imply statistical significance. Abbreviations: EEG, electroencephalography; CN, healthy controls; AD, Alzheimer’s disease; EOR, eyes-open resting state.

In this study, our findings revealed an overall increase in gamma band activity across the 19 EEG channels in AD patients, with notable elevations in the frontal and temporal brain regions during both ECR and EOR conditions (Figure 2b). PSD analysis further demonstrated that gamma power was significantly higher in the AD group compared to the CN group, with the most pronounced difference observed in the ECR condition (Table 1). Statistical analysis confirmed these findings, revealing a significant increase in gamma power from EOR to ECR conditions in both groups (*p* < 0.001). However, the effect size was negligible in the CN group (Cohen’s *d* = 0.0894), whereas the AD group exhibited a markedly large effect (Cohen’s *d* = 1.9000), indicating a more pronounced neural response during ECR in AD. Although unadjusted comparisons showed significantly elevated gamma power in AD relative to CN under both conditions (*p* < 0.001), these group differences did not remain statistically significant after adjusting for age. This attenuation suggests that age may contribute substantially to the observed elevations in gamma activity, underscoring the need to account for age-related neural changes when interpreting group-level differences in PSD. Additionally, pairwise coherence and coherence-based functional connectivity between brain regions were markedly enhanced in the AD group during the ECR (Figure 3b and Figure 5b) but diminished during the EOR state (Figure 5b and Figure 6b) compared to CN participants.

In the EOR condition, Figure 5a (CN) shows lower gamma power and weaker pairwise coherence than Figure 5b (AD), where elevated coherence is particularly prominent in the frontal and temporal regions. This heightened synchronization in the AD group likely reflects compensatory overactivation or pathological network reorganization due to disrupted inhibitory mechanisms [42,43,44], and Figure 6 further highlights these differences, with CN (a) showing evenly distributed gamma power and limited connectivity, indicative of stable neural activity. In contrast, AD (b) exhibits pronounced gamma power increases in the frontal and temporal regions with stronger localized coherence-based functional connectivity. These patterns suggest aberrant network dynamics in AD during EOR. Compared to the ECR condition (Figure 4 and Figure 5), as noted in our results, gamma power and connectivity are significantly higher in AD during ECR, whereas CN demonstrates reduced activity, reflecting balanced neural dynamics.

In the literature, gamma oscillations have been linked to memory, attention, and perception mechanisms, which are commonly disrupted in AD [45]. Despite this, hippocampal atrophy and declines in cognitive performance across domains, such as memory, attention, and perception, have been reported in AD pathology and the aging process [46,47,48]. In addition to these structural and cognitive deficits, our findings revealed significant alterations in gamma band activity in AD patients compared to CN. Specifically, we observed increased gamma power in AD patients across the frontal and temporal scalp regions, particularly during the ECR condition, accompanied by elevated pairwise coherence and coherence-based functional connectivity between cortical areas.

Recent research on gamma oscillations has highlighted the potential of 40 Hz visual and auditory gamma entrainment as a therapeutic approach for AD [49]. By contrast, further findings by Lee et al. [50] suggested that the optimal gamma entrainment frequency may lie within the 34–38 Hz range, mainly when using 100 cd/m^2^ white light in healthy participants. These results indicate the need for further investigation to identify the most effective frequency within the gamma range for entrainment applications, particularly in AD.

Wang et al. investigated alterations in EEG oscillation dynamics in AD patients, focusing on cross-frequency couplings, which play a crucial role in cognition but remain poorly understood in AD. ECR EEG recordings from AD patients and CN revealed increased gamma power in AD patients [9]. The study proposed that the pathological increase in gamma power could stem from GABAergic interneuron network disruptions, a hallmark of AD pathology. These findings align with our study, which also observed increased gamma band activity in AD patients during the ECR condition and alterations in relative PSD compared to CN.

Güntekin et al. examined rEEG gamma activity alterations in AD patients compared to healthy elderly and young individuals, focusing on gamma power values within the 28–48 Hz range. Their study demonstrated that AD patients’ gamma dominant peak frequency was significantly lower than that of healthy elderly and young subjects [13]. Building on these findings, our study investigated relative PSD changes in the gamma frequency band between AD patients and CN using rEEG (EOR and ECR) recordings. By utilizing relative PSD analysis, our approach normalizes gamma activity relative to the total power across all frequency bands, allowing for more standardized and meaningful comparisons between participants and experimental conditions. This novel perspective contributes to a deeper understanding of gamma band dynamics in AD, offering valuable insights into the current neural status of AD patients.

Increased gamma band activity in AD subjects may reflect disruptions in inhibitory interneuron function, such as parvalbumin-positive cells (PV cells), which are vulnerable in AD pathology [8,51,52]. This disruption aligns with our findings and reflects an imbalance in excitatory and inhibitory activities, critical for generating hippocampal gamma oscillations, ultimately resulting in aberrant network activity [53,54]. Our study observed increased gamma power during the ECR condition in AD patients, contrasted with a decrease in CN. This divergence may stem from compensatory overactivation in AD, where impaired inhibitory mechanisms lead to heightened gamma activity as the brain attempts to maintain coherence-based functional connectivity [55,56].

Although gamma power was lower in the CN group, attributing this to more balanced neural dynamics is unwarranted without reference to normative population data. The elevated gamma activity observed in the AD group reached significance in unadjusted analyses but did not persist after adjusting for age, indicating a potential influence of age-related factors. These findings underscore the need for future research incorporating normative baselines and neurophysiological markers, including PV cell dysfunction, to elucidate the mechanisms underlying gamma band alterations in AD.

The variability in outcomes in different research on the gamma frequency band can be attributed to three significant factors. First, differences in the composition of AD patient cohorts, including variations in sex, age, and disease severity as measured by Mini-Mental State Examination (MMSE) scores. Second, there are discrepancies in the types of data collected and the preprocessing techniques applied. Lastly, the selection of distinct frequency bands in the methodological approaches used across studies.

This study focused exclusively on individuals with severe AD in comparison with the CN group, excluding patients with MCI or moderate-stage AD. While our findings suggest that spectral and synchrony features may assist in distinguishing AD patients from healthy controls, it is essential to note that these results are preliminary. The coherence results provide valuable insights into large-scale neural synchronization; however, these analyses were conducted at the sensor level using a 19-channel EEG system, which inherently constrains spatial resolution and precludes precise anatomical localization of underlying neural generators. Furthermore, resting-state gamma activity is inherently variable and has been regarded as a less reliable biomarker [57]. To address this, we applied robust preprocessing techniques, including artifact removal and signal correction, to enhance data quality.

In future work, we intend to incorporate stimulus-induced gamma oscillations, which offer greater specificity and reliability [58]. We also plan to use high-density EEG and advanced source reconstruction techniques to improve anatomical precision and the interpretive validity of functional connectivity analyses. Additionally, our analysis will be extended to include a broader range of dementia subtypes and disease stages. Integrating resting-state EEG findings with gamma-frequency entrainment paradigms such as 40 Hz visual or auditory stimulation may also yield valuable insights into the therapeutic potential of gamma modulation in AD. While preliminary preclinical and clinical studies have shown promising effects on neural synchrony, amyloid pathology, and neuroimmune function [59,60,61,62,63,64], the relationship between endogenous gamma activity and entrainment responsiveness remains poorly understood. Future studies should explore whether baseline gamma PSD characteristics can predict therapeutic outcomes and support personalized intervention strategies in dementia care.

## 5. Conclusions

This study underscores the utility of rEEG gamma band analysis as a valuable tool in understanding AD progression. Our findings revealed elevated gamma power and heightened coherence-based functional connectivity in the AD group compared to CN, particularly in the frontal and temporal brain regions during both EOR and ECR conditions, with the most pronounced differences observed in ECR. The results demonstrate that the gamma frequency band (30–100 Hz) is a promising biomarker for distinguishing AD patients from CN, highlighting its potential for early and accurate diagnosis of dementia. These findings align with recent research supporting the use of gamma band activity in dementia studies and emphasize its cost-effectiveness and applicability in clinical settings. By leveraging rEEG data and established analytical approaches, including relative PSD and coherence analyses, this study contributes to the growing body of evidence on the critical role of gamma oscillations in AD pathology. Yet, age-adjusted analyses indicated that the observed gamma differences were no longer statistically significant, suggesting that age-related factors may partially account for the group differences. Future research should focus on integrating these insights into clinical practices, paving the way for improved diagnostic accuracy and timely interventions in dementia care.

## Figures and Tables

**Table 1:** Within-group comparison of relative gamma band PSD between EOR and ECR conditions in CN and AD groups.

Group	EOR (Mean ± SD)	ECR (Mean ± SD)	*p*-Value	Cohen’s *d*
CN (*n* = 269)	0.0040 ± 0.0030	0.0042 ± 0.0010	<0.001	0.0894
AD (*n* = 265)	0.0060 ± 0.0080	0.0860 ± 0.0590	<0.001	1.9000

Note: Values are presented as the mean ± standard deviation (SD). Abbreviations: PSD, power spectral density; CN, cognitively normal; AD, Alzheimer’s disease; EOR, eyes-open resting state; ECR, eyes-closed resting state.

**Table 2:** Comparison of gamma band power between CN and AD in unadjusted and age-adjusted analyses.

Condition	Model	Group/ Comparison	Gamma PSD (Mean ± SD)	β (Adjusted)	SE	*p*-Value
EOR	Unadjusted	CN	0.0040 ± 0.0030			<0.001
		AD	0.0060 ± 0.0080			<0.001
	Adjusted	CN vs. AD		−0.0022	0.0033	0.504
		Age		−0.0003	0.0002	0.076
ECR	Unadjusted	CN	0.0042 ± 0.0010			<0.001
		AD	0.0860 ± 0.0590			<0.001
	Adjusted	CN vs. AD		−0.0047	0.0054	0.391
		Age		−0.0008	0.0003	0.019

Note: Unadjusted values are reported as the mean ± standard deviation (SD). Abbreviations: PSD, power spectral density; β, beta coefficient; SE, standard error; CN, cognitively normal; AD, Alzheimer’s disease; EOR, eyes-open resting state; ECR, eyes-closed resting state.

## Data Availability

The data supporting the findings of this study are available upon reasonable request from the corresponding author Y.C.Y via email. Due to confidentiality agreements and institutional policies protecting sensitive patient information from the hospital where the study was conducted, the data cannot be made publicly available. Requests will be evaluated to ensure compliance with ethical and legal standards.

## Supplementary Materials

The following supporting information can be downloaded at: https://www.mdpi.com/article/10.3390/jcm14124256/s1, Figure S1: 19 Scalp Electrodes Positioned based on the International 10–20 System; Table S1: Components and Scores of SNSB; Table S2: Gamma Band PSD Values by Channel, Group, and Condition (EOR vs. ECR).
