# Supplementary material for: Investigating Gamma Frequency Band PSD in Alzheimer’s Disease Using qEEG from Eyes-Open and Eyes-Closed Resting States
Source: J Clin Med. 2025 Jun 15;14(12):4256. doi: 10.3390/jcm14124256 (PMC12194015; doi:10.3390/jcm14124256)

Supplementary Table S1 Components and Scores of SNSB

Domains	Score	%	Subtests	Maximum points
Attention	17	6	Digit span forward<br>Digit span backward	9<br>8
Language and related function	27	9	Short form of K-BNT (A form)<br>Calculation<br>(3 items each for addition, Subtraction, multiplication, division)	15<br>12
Visuospatial function	36	12	RCFT copy	36
Memory	150	50	Orientation<br>SVLT free/delayed recalls<br>SVLT recognition<br>RCFT immediate/delayed recalls<br>RCFT recognition	6
Frontal/Executive function	70	23	Motor impersisitence<br>Contrasting program<br>Go-no-go test<br>Fist-edge-palm<br>Luria loop<br>Category word generation<br>(animal)	3<br>3<br>3<br>3<br>3<br>20<br>15

			Phonemic word generation<br>Stroop test-color reading	20
GCF score	30	100		

Abbreviations: K-BNT, Korean-Boston naming test; RCFT, Rey-complex figure test; SVLT, Seoul verbal learning test; GCF, global cognitive function.

Supplementary Table S2 Gamma Band PSD Values by Channel, Group, and Condition (EOR vs. ECR)

Channel	Group	Condition	Gamma PSD
Fp1	CN	EOR	8.19E-14
Fp1	CN	ECR	1.08E-13
Fp1	AD	EOR	2.06E-13
Fp1	AD	ECR	2.61E-13
F3	CN	EOR	6.19E-14
F3	CN	ECR	2.29E-14
F3	AD	EOR	1.39E-13
F3	AD	ECR	4.34E-14
C3	CN	EOR	2.35E-14
C3	CN	ECR	9.98E-15
C3	AD	EOR	1.13E-13
C3	AD	ECR	7.63E-14
P3	CN	EOR	2.37E-14
P3	CN	ECR	1.34E-14
P3	AD	EOR	2.01E-14

P3	AD	ECR	2.09E-14
O1	CN	EOR	3.54E-14
O1	CN	ECR	1.46E-14
O1	AD	EOR	2.01E-14
O1	AD	ECR	2.79E-14
Fp2	CN	EOR	2.40E-13
Fp2	CN	ECR	1.56E-13
Fp2	AD	EOR	2.07E-13
Fp2	AD	ECR	3.38E-13
F4	CN	EOR	2.82E-14
F4	CN	ECR	8.14E-14
F4	AD	EOR	1.01E-13
F4	AD	ECR	1.91E-13
C4	CN	EOR	2.76E-14
C4	CN	ECR	1.28E-14
C4	AD	EOR	9.55E-14
C4	AD	ECR	5.47E-14
P4	CN	EOR	3.85E-14
P4	CN	ECR	2.01E-14
P4	AD	EOR	2.61E-14
P4	AD	ECR	2.58E-14
O2	CN	EOR	4.40E-14
O2	CN	ECR	2.10E-14
O2	AD	EOR	2.81E-14

O2	AD	ECR	3.22E-14
F7	CN	EOR	2.23E-14
F7	CN	ECR	1.04E-14
F7	AD	EOR	7.00E-14
F7	AD	ECR	8.17E-14
T3	CN	EOR	3.07E-14
T3	CN	ECR	1.43E-14
T3	AD	EOR	1.52E-13
T3	AD	ECR	1.67E-13
T5	CN	EOR	2.48E-14
T5	CN	ECR	1.79E-14
T5	AD	EOR	3.29E-14
T5	AD	ECR	4.38E-14
F8	CN	EOR	2.66E-14
F8	CN	ECR	2.94E-14
F8	AD	EOR	5.18E-14
F8	AD	ECR	1.82E-13
T4	CN	EOR	4.57E-14
T4	CN	ECR	1.78E-14
T4	AD	EOR	1.96E-13
T4	AD	ECR	1.84E-13
T6	CN	EOR	3.19E-14
T6	CN	ECR	3.47E-14
T6	AD	EOR	3.32E-14

T6	AD	ECR	4.13E-14
Fz	CN	EOR	3.48E-14
Fz	CN	ECR	3.60E-14
Fz	AD	EOR	3.56E-14
Fz	AD	ECR	5.62E-14
Cz	CN	EOR	2.47E-14
Cz	CN	ECR	1.14E-14
Cz	AD	EOR	2.73E-14
Cz	AD	ECR	2.37E-14
Pz	CN	EOR	3.08E-14
Pz	CN	ECR	1.55E-14
Pz	AD	EOR	2.17E-14
Pz	AD	ECR	2.22E-14

Abbreviations: PSD; Power Spectrum Density; EOR, Eyes-Open Resting State; ECR, Eyes-Closed Resting State; CN, Cognitively Normal; AD, Alzheimer's Disease.

Supplementary Figure S1 19 Scalp Electrodes Positioned based on the International 10-20 System

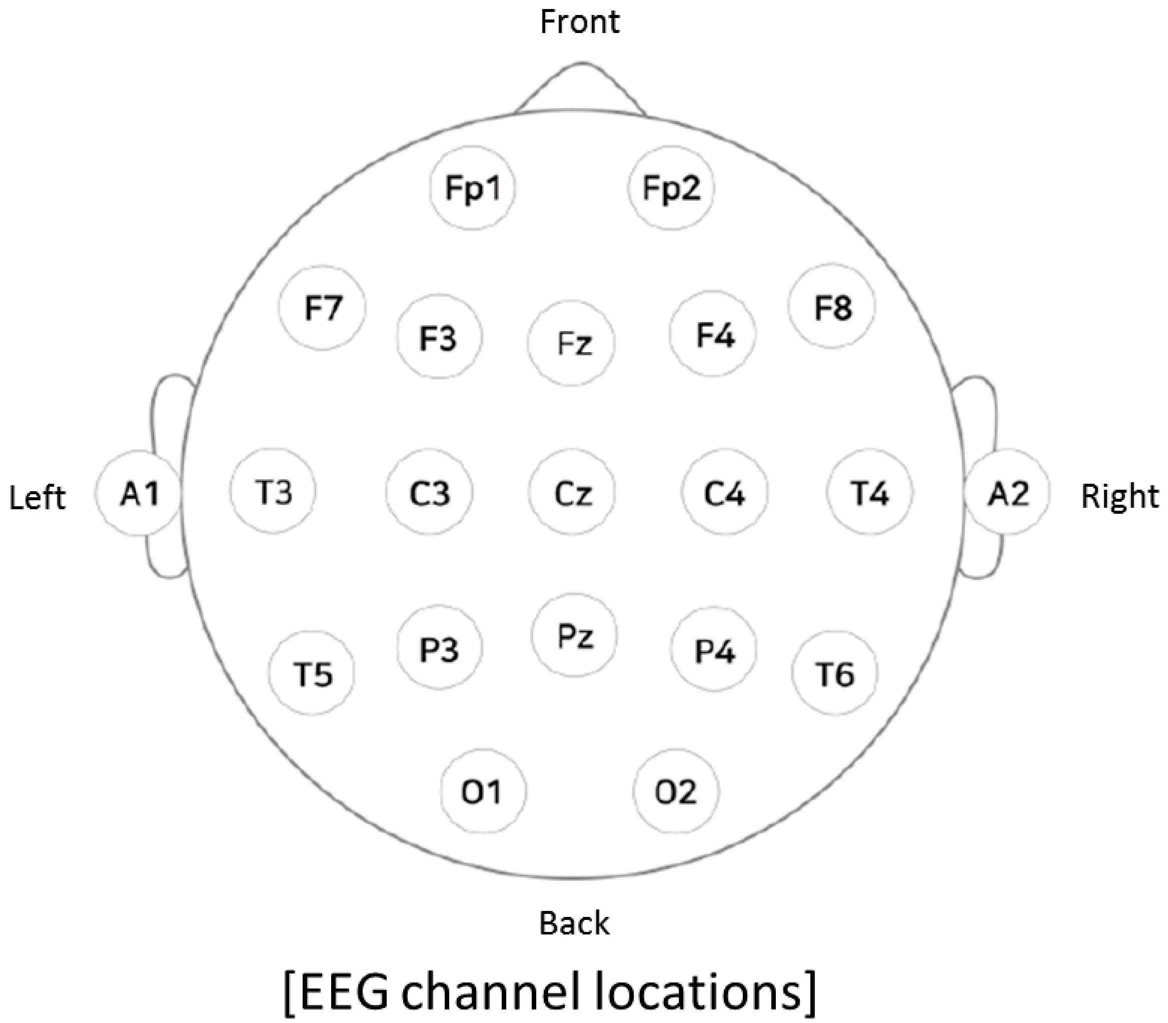

Supplement: Supplementary file 1 [file jcm-14-04256-s001.zip › jcm-3664925-supplementary.pdf]
